# Development and Validation of the Large Language Model Privacy Concern Assessment Questionnaire: Psychometric Evaluation Study on a Brief Instrument for Assessing User Privacy Concerns With Large Language Model Applications

**DOI:** 10.2196/102910

**Published:** 2026-10-21

**Authors:** Leming Zhou, Hongtao Wang, Yi Xu, I Made Agus Setiawan, Haomin Hu, Andi Saptono, Yong K Choi, Bambang Parmanto

**Affiliations:** 1 Department of Health Information Management, School of Health and Rehabilitation Sciences, University of Pittsburgh, 3396 Fifth Avenue, Pittsburgh, PA, 15213, United States, 1 412-383-6653

**Keywords:** privacy, questionnaire, psychometric analysis, large language models, scale validation

## Abstract

**Background:**

Privacy concerns represent a primary barrier to the adoption of large language model (LLM) applications, particularly in sensitive domains such as health care. Although a growing body of survey-based research has examined user privacy concerns with LLMs, no standardized instrument exists for measuring this construct.

**Objective:**

This study aimed to develop and validate a concise, standardized questionnaire for assessing user privacy concerns with LLM-based systems, suitable for use by researchers and practitioners such as AI application developers and health care professionals.

**Methods:**

A literature review was conducted to identify privacy-related domains relevant to LLM use. A draft questionnaire was developed and subjected to content validity review by 8 experts. The finalized instrument was administered via Qualtrics to adults aged 18 years or older who were aware of LLM-based applications, recruited through the University of Pittsburgh, LinkedIn, and the Pitt+Me research participant registry between April 2025 and July 2026. Internal consistency reliability and factor structure were assessed, with factor structure examined in a split-sample design, in which exploratory factor analysis (EFA) was conducted on a random one-third of the sample and confirmatory factor analysis (CFA) on the holdout two-thirds.

**Results:**

All questionnaire items achieved acceptable content validity (item-level content validity index >0.80; average scale-level content validity index=0.964). A total of 603 responses were collected. After preprocessing, the final sample comprised 361 participants. Most participants were younger than 35 years of age (198/361, 54.8%), female (230/361, 63.7%), and White (285/361, 78.9%); 88.1% (318/361) held at least a bachelor’s degree. The 6-item questionnaire showed high internal consistency (Cronbach α=0.90; McDonald ω=0.90). EFA (n=121) supported a single dominant factor with all standardized loadings ≥0.70. CFA (n=240) showed excellent fit (comparative fit index=0.996; Tucker-Lewis index=0.993; root mean square error of approximation=0.046; standardized root mean square residual=0.057). A 2-factor model separating the transparency- and control-related items fit significantly better than the 1-factor model (Δ*χ*^2^_1_=8.9; *P*=.003), but the 2 factors were correlated at *r*=0.83, indicating closely related facets. Because the Fornell-Larcker criterion was met for the transparency and control subscale but not for data use and risk salience, the 6-item total score remains the recommended primary metric.

**Conclusions:**

The LLM privacy concern assessment is a concise, psychometrically sound 6-item instrument that measures privacy concern with LLM-based systems via 2 correlated subdimensions (transparency and control; data use and risk salience) summarized by a single total score. It enables standardized measurement across studies and practical privacy assessment in real-world LLM applications.

## Introduction

### Background

Large language models (LLMs) have seen rapid and widespread adoption across domains ranging from general-purpose information retrieval to specialized health care consultations. As users increasingly engage with these systems for sensitive tasks, including health queries, financial decisions, and personal problem-solving, privacy concerns have emerged as a primary barrier to adoption and sustained use [1-3]. These concerns are well-founded because user-entered data often include highly sensitive information about health, finances, and personal relationships that could be used in the next round of LLM training. Unlike traditional regulated health platforms, most general-purpose LLMs currently operate outside established comprehensive data protection frameworks, leaving users with limited assurances regarding how their information is handled [4].

Information privacy concern has been investigated since the 1990s through a succession of instruments, each linked to the development of information technology and responding to the data practices of its era [5]. The Concern for Information Privacy (CFIP) scale of Smith et al [6] framed privacy concern as apprehension about organizational data handling, with 4 domains addressing collection, unauthorized secondary use, improper access, and errors. The Internet Users’ Information Privacy Concerns (IUIPC) scale of Malhotra et al [7] recasts concern as an appraisal of the fairness of a data exchange, drawing on social contract theory and measures collection, control, and awareness, while the Mobile Users’ Information Privacy Concerns (MUIPC) scale of Xu et al [8] draws on communication privacy management theory and measures perceived surveillance, perceived intrusion, and secondary use. Later instruments pursued either integration, nesting several first-order domains under a higher-order general factor, as in Hong and Thong’s Internet Privacy Concerns (IPC) scale [9], or reducing the construct to a small number of platform-specific domains, as in the scale of Sheehan and Hoy [10] for online consumers and in the scale of Wang et al [11] for short-form video platforms. Across this lineage, a core recurs, namely, collection, awareness, and use or reuse of data, while the remaining domains and the dimensional structure itself shift with the referent, from organizational record-keeping to the commercial web and then to mobile and social media platforms [6-11], and each shift left the preceding generation of items a poorer fit than it had been. That fragility is not only conceptual but linguistic. Item-interpretation studies show that widely used privacy statements often measure more than 1 construct and are read differently by different populations [12,13], and re-examinations on independent data have repeatedly led to respecification of published factor structures and revealed validity and reliability shortfalls [14,15]. This lineage demonstrates that privacy concern instruments are context-bound. Each of these instruments was created because a shift in technology, referent, theory, or population made prior items ill-fitting. For any new instrument, the dimensional structure, item wording, and cross-context validity must be established rather than assumed.

LLM-based applications meet that same test on more than 1 dimension, which is the basis for developing a new instrument rather than adopting an existing one. First, the referent itself differs, because prior scales address an organization, website, or platform, whereas many users often interact with an LLM as a conversational partner and disclose information in natural language without a clear idea about how their inputs are stored and reused [16]. Second, the data practices also differ. User inputs may be retained for model training, and LLMs can memorize and later reproduce fragments of their training data [17] or infer unstated personal attributes such as employment, location, income, and sex from ordinary text, which is an inferential privacy risk with no close analogue in form-based web interaction [18]. The LLM provider ecosystem is also opaque and multiparty, so retention, sharing, and reuse of data are largely unobservable to the user. Finally, because item meaning does not transfer cleanly across referents [12,13], even a shared construct such as data “collection” is unlikely to be measurement-invariant when the collecting entity is a generative AI model rather than a website.

A first wave of survey studies has begun to measure users’ privacy concerns with LLMs and AI chatbots, several of them in health contexts, but each has operationalized privacy through a distinct theoretical lens and for a distinct purpose. Choi et al [1] assessed disclosure willingness and its antecedents across 3 countries using items derived from the antecedents, privacy concerns, and outcomes (APCO) macromodel [5]; Liu et al [2] used privacy concern as a predictor within a behavioral model grounded in uncertainty reduction theory [19]; Liu et al [3] measured privacy awareness and expectation gaps using information flows informed by contextual integrity [20]; and Tran et al [4] used factorial vignettes to assess the contextual conditions under which data sharing is perceived as appropriate. Although each of these instruments provides theoretically rich insights within its specific framework, none was designed to yield a generalizable, stand-alone privacy concern score that could be compared across studies or applied by practitioners to a new LLM system.

What is missing is a validated, purpose-built measure of user privacy concern toward LLMs that is not tied to any single behavioral theory or downstream outcome, and that a researcher or LLM application developer can apply directly to assess privacy concern toward a specific system. This gap motivates the development of a concise, referent-specific instrument that preserves the field’s replicated conceptual core, reanchors it to the LLM context, and extends it to concerns unique to conversational AI.

### Objectives

The goal of this study is to develop and validate a concise, standardized instrument for assessing user privacy concerns specific to LLM-based systems. The instrument is intended to serve both researchers who need a reusable scale enabling cross-study comparison and practitioners who wish to conduct privacy assessments of specific LLM applications. Development followed a multistage process including item creation informed by the literature, expert content validation, and psychometric evaluation in a general adult sample.

## Methods

### Survey Development

Two authors in the study team (LZ and HW) conducted a literature review to identify privacy domains relevant to LLM use, including technology vendor trust, informed consent, data handling practices, user control, and transparency. Based on this review, a draft questionnaire was developed. A literature search was performed on Google Scholar, PubMed, and Scopus using keywords [“privacy concern” AND (questionnaire OR instrument OR scale) AND (internet OR web OR e-commerce OR “mobile app” OR “short form video” OR “Artificial Intelligence” OR AI)] without date restrictions. LZ and HW worked together and selected articles on questionnaire development and the ones that have been widely adopted in other studies. Some articles were identified from the references of selected articles. We ignored articles that simply applied these questionnaires to measure privacy concern or presented a language translation of them. The domains and questionnaire items from the 6 selected articles [6-11] were extracted and compared against the uniqueness of privacy concerns with LLMs (eg, the opacity of model vendors, new ways of providing personal data, and new ways of reusing user data).

Candidate domains were identified from the comparative synthesis of the 6 foundational instruments and the LLM privacy literature, following recommended practice of grounding a construct’s content in theory and prior measurement before item generation [21]. We retained the domains with the strongest cross-instrument replication (data use, awareness, and control) since they were still highly relevant to privacy concerns with LLM applications, and added domains warranted by the distinctive data practices of LLMs (transparency for trust, risk salience, and onward third-party sharing), plus a global-concern domain to support an overall index.

Theoretically, these candidate domains are consistent with the APCO framing of privacy concern as a central antecedent of privacy outcomes [5], with social contract accounts of fairness and control in data exchange [7,22], and with communication privacy management theory’s emphasis on control over disclosure boundaries [8]. All of these are strained in the conversational, inference-capable LLM setting. Grounding the domains in this measurement lineage rather than in any single behavioral theory is deliberate because recent LLM questionnaires built on APCO, uncertainty reduction theory, and contextual integrity each measured a different facet of concern and produced noncomparable scores [1-4], so this new questionnaire specifies its content at the construct level to remain usable across those frameworks.

The initial working draft included more than 1 candidate item for 7 domains; through internal discussion, and before the independent content-validity review, the pool was reduced to 1 item per domain. Wording followed the lessons of the interpretation-and-validity literature [12,13]: items are short and adopt a single consistent perspective, that is, the person’s own concern or valuation, rather than mixing concern-perception with expectation phrasing [9]. Each item names a referent (“LLMs” or “LLM-based applications”) so that responses attach to the intended entity rather than to websites or platforms generally, directly addressing the referent-mismatch problem the validity studies document. Response formats use ordinal Likert-type intensity anchors appropriate to each stem (eg, degree of concern, degree of importance, and frequency).

The instrument is intended as a brief privacy-concern index, scored as a single composite and composed of 1 item per narrowly defined domain, rather than a multi-item-per-factor multidimensional battery. This is a deliberate design choice, not a claim that privacy concern is psychologically unidimensional. Where a construct or domain is concrete and singular, single-item measures can perform comparably to multi-item scales and are defensible on both predictive validity and practical grounds [23-25]. A one-item-per-domain design maximizes content breadth across the privacy space while minimizing respondent burden and the wording-induced interpretation problems that multi-item scales compound [12,13]. The trade-offs, no within-domain internal-consistency estimate and greater sensitivity to item wording, are mitigated by the validation plan below and by the deliberate, theory-anchored specification of each domain. We acknowledge these limitations in the Discussion section, and we regard the instrument as preliminary. To avoid a terminological ambiguity, we note that the measurement model estimated in this study is reflective: each item is treated as an effect indicator of a common latent privacy-concern factor, which justifies the use of factor-analytic and internal-consistency evidence [26].

Responses were measured on a 5-point Likert scale with anchors tailored to each item’s stem: importance items were anchored 1=extremely important to 5=not at all important; concern items were anchored 1=extremely concerned to 5=not at all concerned; and the frequency item was anchored 1=always to 5=never. The use of heterogeneous anchors (importance, concern, and frequency) was intentional to match the conceptual content of each item; the subsequent factor analysis tested whether items cohere as a single scale despite the differing anchors. All 3 anchor sets run in the same direction, from the strongest to the weakest degree of concern, so lower raw scores indicate stronger privacy concern, and item scores should be reverse-coded before a composite is formed if higher scores are to denote greater concern. The composite is a unit-weighted sum or mean of the 6 raw item responses, which assumes that the 5 ordinal positions are metrically equivalent across the 3 anchor types. The present data cannot verify that assumption. The items are known only to share a common 5-category format and common direction. We retained unit weighting rather than standardizing items to *z* scores for 2 reasons: unit-weighted composites are generally as predictively useful as differentially weighted ones and more stable across samples [27]; *z* standardization would make the score dependent on the composition of the sample in which it is computed, which is unsuitable for the benchmarking use for which the instrument is intended. Two questions are therefore left open: whether the 3 anchor types function equivalently across respondents, and whether unit-weighted and standardized scoring lead to the same conclusions. Both are discussed in the Limitations and Future Work section.

Validation followed established scale-development guidance [21,28]. Content validity of the questionnaire was evaluated by a panel of 8 independent subject-matter experts. To preserve the independence of the appraisal, the panel comprised only experts who were not involved in item construction. They were recruited via research team members’ recommendations. Their backgrounds were computer science, information science, and user experience research, and all had extensive experience in questionnaire design and evaluation. Each expert independently rated every item on 2 dimensions, relevance to the target construct (user privacy concern with LLM applications) and clarity of wording, using the 4-point ordinal scale recommended by Lynn [29]: 1=not relevant or clear, 2=somewhat, 3=quite, and 4=highly relevant or clear. Ratings of 3 or 4 were recoded as endorsement and ratings of 1 or 2 as nonendorsement [29,30]. Items for which the majority of reviewers assigned a relevance rating of 1 or 2 were removed. Items receiving low clarity ratings were retained but revised through team consensus during a structured review meeting. For each item, the item-level content validity index (I-CVI) was computed as the proportion of experts who endorsed the item. Because the I-CVI=*A*/*N* does not adjust for chance agreement, a modified kappa (κ*) was also calculated by correcting each I-CVI for the probability of chance agreement, *P*_C_=[*N*!/(*A*!(*N*−*A*)!)](0.5^*N*^), where *N* is the number of experts, and *A* is the number endorsing the item; κ*=(I-CVI–*P*_C_)/(1–*P*_C_) was interpreted as excellent (>0.74), good (0.60-0.74), or fair (0.40-0.59) [31]. At the scale level, both the averaging method (scale-level content validity index [S-CVI]/Ave and the mean of the item I-CVIs) and the universal-agreement (UA) method (S-CVI/UA, the proportion of items endorsed by all experts) were reported [30]. An I-CVI of at least 0.78 for panels of 6 or more experts and an S-CVI/Ave of at least 0.90 were adopted a priori as evidence of satisfactory content validity [29-31]. Structural validity was assessed with exploratory factor analysis (EFA) followed by confirmatory factor analysis (CFA), evaluated against conventional fit criteria [32]. Reliability was reported as internal consistency and composite reliability where applicable. Further details are provided in the Statistical Analysis section.

The panel’s ratings are one component of the content validity evidence rather than the whole of it. The theoretical grounding of the candidate domains and the mapping of each item to its domain and to the prior constructs from which that domain derives are reported in Table 1. Because the panel comprised 8 reviewers recruited on the recommendation of research team members rather than by independent nomination, its endorsement does not by itself establish content validity. The size of the panel is also small. Both are noted in the Limitations and Future Work section.

**Table 1.** Mapping between proposed questionnaire domains and constructs in prior instruments.

LLM-PCA^a^ item	Domain	Antecedent constructs in prior instruments	Primary lineage source
G1. How concerned are you about the ways in which LLMs^b^ use your personal data?	Data use (secondary/onward use)	Unauthorized secondary use, information use, use concerns	[6-9,11]
G2. How important is it for you to be informed about how LLMs use your data?	Awareness (notification)	Awareness of collection, awareness	[7,9,10]
G3. How much concern do you have for the privacy of your data when using applications or services that utilize LLMs?	Global privacy concern	Overall or general privacy-concern factor	[7,9]
G4. How important is transparency about data usage to your trust in LLM-based applications?	Transparency (trust-relevant)	Awareness, link to trusting beliefs	[7,9]
G5. How important is it for you to have control over how LLMs use your data?	Control	Control	[7,9]
G6. How often do you think about the potential privacy risks when using LLM-based applications?	Risk salience	Link to risk beliefs	[9]
G7. How important is it for you to know about the specific companies or organizations with whom your data is shared?	Third-party or onward sharing	Unauthorized secondary use, improper access, perceived surveillance, third-party sharing within use concerns	[6,8,9,11]

a LLM-PCA: large language model privacy concern assessment.

b LLM: large language model.

The revised instrument was then deployed on Qualtrics (Qualtrics International) for an internal pilot test conducted with the full research team. The pilot assessed the question-and-response logic, item flow, and platform implementation. Issues identified during the pilot were resolved prior to participant recruitment. In addition to the privacy-concern items, the survey collected demographic information, including age, race, sex, education level, marital status, and annual income, to assess potential biases in respondents’ answers, plus one screening question to determine whether potential participants had heard of LLMs. If they answered no or did not provide a response to this screening question, they would not be allowed to answer any questions on privacy concerns with LLMs. Responses from individuals unfamiliar with LLMs would not meaningfully reflect privacy concerns about them. The entire procedure is shown in Figure 1.

**Figure 1.**
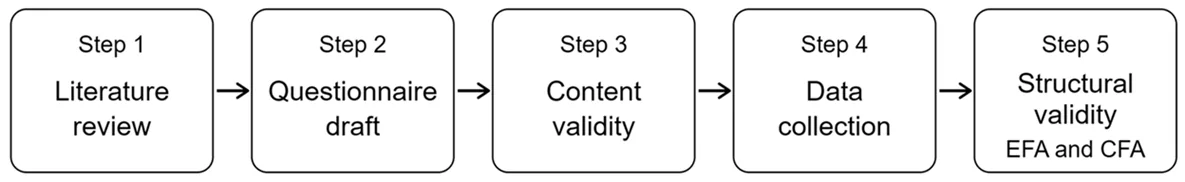
Flowchart of questionnaire development and validation. CFA: confirmatory factor analysis; EFA: exploratory factor analysis.

### Participant Recruitment

Participants were eligible if they were adults (aged ≥18 years), understood English, and had awareness of LLM-based applications. Recruitment was conducted through three channels: (1) bulk email distributed to a random sample of students, faculty, and staff affiliated with the University of Pittsburgh; (2) a posting on LinkedIn; and (3) a posting on Pitt+Me, a research participant registry managed by the University of Pittsburgh. Interested individuals accessed the survey via a link directing them to the Qualtrics platform. When the university’s electronic bulk-mail delivery service received a request for an announcement, it randomly selected the targeted audience (faculty, students, and staff in this case) and distributed the announcement. We used the service and made 2 announcements about the study in April and May 2025. In total, the message was delivered to several thousand faculty, students, and staff at Pitt. Study participation was fully voluntary, and we did not pay any compensation to the study participants. The same recruitment message was posted on the website of Pitt+Me in April, and anyone who visited the website in the study period (April 2025 to July 2026) could see the recruitment message and follow the link to answer the questions in the questionnaire.

### Ethical Considerations

This study was approved by the University of Pittsburgh Human Research Protection Office (protocol ID: STUDY24110036). Participation was entirely voluntary; participants could withdraw at any time and request removal of their data without penalty.

### Data Preprocessing

Prior to analysis, the raw dataset was cleaned using the following sequential criteria. First, records collected during the internal pilot phase were excluded. Second, responses that were entirely blank and responses that contained only demographic answers with no answers to any privacy concern item were removed as nonparticipating records because these entries could not provide any information for the privacy concern questionnaire. Third, participants who indicated that they had never heard of LLMs were excluded, as responses from individuals with no awareness of the technology were considered uninformative for the study’s objectives. They were not allowed to answer any further questions in the questionnaire. Fourth, participants who did not answer any privacy concern–related questions were excluded since their data would not be useful for this specific study. These 4 steps removed records that should not be included in the dataset. The fifth step was a decision about how to handle missing data. We chose to only keep records with complete answers to those privacy concern questionnaire items. We adopted complete-case analysis because no imputation approach is free of assumptions, and imputed values could alter the item distributions on which the psychometric analyses depend. Records missing demographic data only were retained since they would not impact the evaluation of the large language model privacy concern assessment (LLM-PCA) questionnaire itself. Finally, administrative columns (eg, response ID, timestamp, and language) were removed from the dataset.

To evaluate whether restricting analyses to complete cases introduced systematic bias, we compared respondents retained for complete-case analysis with those excluded due to partial item nonresponse across all study variables. Continuous variables were compared using independent samples *t* tests, and categorical variables using Pearson chi-square tests of independence. A 2-sided α of .05 was applied. Because these tests were intended to detect potential nonresponse bias rather than to establish statistical equivalence, nonsignificant differences were interpreted as consistent with, but not confirmation of, ignorable missingness.

### Statistical Analysis

Descriptive statistics were computed for all demographic variables, with frequencies and percentages reported. Item-level descriptive statistics (mean, SD, count of respondents to each option, and percentage) were computed for each item in the privacy concern questionnaire prior to factor analysis. Psychometric analyses were then conducted on the privacy concern items. Internal consistency reliability was assessed using Cronbach α and McDonald ω, with values ≥0.70 considered acceptable [33,34].

To examine the dimensionality and measurement properties of the scale, we used a split-sample cross-validation design: the complete-case sample was randomly partitioned into 2 parts, with roughly one-third used for EFA and the remaining two-thirds reserved for CFA. Because the total sample was modest for a split-sample design, we treat the EFA as exploratory and hypothesis-generating; confirmatory replication in a larger and independent sample remains necessary, and the reported CFA fit should be interpreted accordingly [32,34].

Because the items were measured on a 5-point Likert scale, we computed a polychoric correlation matrix to characterize interitem association. Polychoric correlations model an underlying continuous latent response for each ordered-categorical indicator and are the principled measure of association for Likert items. Sampling adequacy was evaluated using the Kaiser-Meyer-Olkin (KMO) measure and Bartlett test of sphericity, both computed from the polychoric matrix. The number of factors to retain was determined by parallel analysis with 500 iterations of resampled data, using the 95th percentile of simulated eigenvalues as the cutoff [35]; Kaiser’s eigenvalue-greater-than-one criterion was reported as a secondary index. Factor extraction used maximum likelihood (ML) on the polychoric correlation matrix; when more than one factor was retained, promax (oblique) rotation was applied. ML extraction on a polychoric matrix assumes multivariate normality of the underlying continuous responses, an assumption we adopted as reasonable for ordinal Likert indicators.

A CFA model with all retained items loading on a single latent privacy concern factor was fitted to the CFA subsample using diagonally weighted least squares (DWLS), the appropriate estimator for ordered-categorical Likert indicators. The latent factor variance was fixed to 1.0 to identify the model, allowing all factor loadings to be freely estimated.

Model fit was evaluated at both the global and local levels because satisfactory global fit does not guarantee that a model reproduces every element of the observed covariance structure. Global fit was assessed using the model chi-square statistic with its degrees of freedom and associated *P* value, the comparative fit index (CFI), the Tucker-Lewis index (TLI), the root mean square error of approximation (RMSEA) with its 90% CI, the standardized root mean square residual (SRMR), goodness-of-fit index (GFI), and adjusted goodness-of-fit index (AGFI). The GFI and AGFI are reported for completeness only and are not used to support any conclusion about fit. Conventional cutoffs for acceptable fit were CFI and TLI ≥0.90 (≥0.95 for good fit), RMSEA ≤0.08 (≤0.06 for good fit), and SRMR ≤0.08 (≤0.05 for good fit). These values were treated as descriptive heuristics and not as decision thresholds. They were derived under normal-theory ML with continuous data [32], and simulation work shows that diagonally weighted and unweighted least squares applied to polychoric matrices yield systematically smaller RMSEA and larger CFI and TLI values than ML, so that applying the conventional cutoffs under these estimators tends to understate misfit [36]. Because the model chi-square test has limited power to detect misspecification at moderate sample sizes and the RMSEA is estimated with appreciable sampling variability, we additionally evaluated close fit using the test of close fit (PCLOSE [*P* value for the test of close fit]), which assesses the null hypothesis that the population RMSEA is no greater than .05. Because the model was estimated with DWLS on the polychoric correlation matrix, particular interpretive weight was given to the SRMR, which is the most robust and least biased of the common fit indices under this estimator and ordered-categorical data. The RMSEA was interpreted together with the width of its CI. Local fit was examined through the matrix of residual correlations, computed as the difference between the observed polychoric correlations and the correlations implied by the fitted model. Individual residual correlations were inspected to identify item pairs whose association the model failed to reproduce; absolute residual correlations greater than 0.10 were treated as indicators of localized strain warranting further examination [37]. The magnitude and pattern of the largest residuals were reported to characterize the nature of any local misfit, and their substantive interpretation was considered alongside the global indices in evaluating the adequacy of the model. Global fit was therefore interpreted jointly with this local fit evidence and with the nested model comparison, rather than by asking whether any single index cleared a cutoff.

Composite reliability (ω) was reported as an indicator of internal consistency, and the average variance extracted (AVE) as an indicator of convergent validity. Where a model with more than 1 factor was retained, discriminant validity between factors was assessed using the Fornell-Larcker criterion [38], which requires the AVE for a factor to exceed the squared correlation between factors, and the heterotrait-monotrait ratio of correlations (HTMT)<0.90 because values of HTMT above 0.90 indicate that factors are not empirically distinct. Reliability and AVE were computed for the full item set and for the indicators defining each factor in any multifactor solution. Composite reliability was computed as ω=(Σλᵢ)^2^/[(Σλᵢ)^2^+Σ(1−λᵢ^2^)], and AVE as AVE=(Σλᵢ^2^)/*k*, where λ*ᵢ* is the standardized loading of indicator i, and *k* is the number of indicators. Conventional thresholds of ω≥0.80 and AVE ≥0.50 were used [34,38].

To assess sensitivity to the random partition, the full procedure, including the item-refinement step, exploratory analysis, and confirmatory analysis, was repeated under 5 random partitions, with all item-reduction decisions made using the exploratory subsample only.

Analyses were conducted in SPSS (version 31; IBM Corp) and Python (version 3.13; Python Software Foundation) using the *pyreadstat* package for SPSS file input, *factor_analyzer* for EFA, and *semopy* for CFA [39]. Path diagrams were first visualized using semopy.semplot and postprocessed for publication.

### Sample Size Considerations

Minimum sample size was estimated during participant recruitment from the general rule of thumb of 10 respondents per item for factor analysis. No formal a priori power analysis was conducted, so recruitment remained open for the duration of the fielding period, and the achieved sample was determined by the response to the 3 recruitment channels. The benchmarks set out below are the a priori criteria, against which the planned analyses were judged to be adequately powered. Whether the achieved subsamples met those benchmarks and whether the data satisfied the conditions the benchmarks assume are addressed in the Results and Limitations and Future Work sections.

For the EFA, the required sample size is not fixed by absolute N or by the subject-to-variable ratio alone but depends primarily on the level of communality and the degree to which factors are overdetermined [40]. When communalities are high and a single factor is well defined by multiple indicators, population factors can be recovered accurately even in small samples. de Winter et al [41] similarly demonstrated that well-conditioned data, characterized by high loadings, few factors, and an adequate number of indicators per factor, yield reliable EFA solutions at sample sizes well below conventional minimums. Neither condition can be verified before the data are collected, so they were treated as criteria to be checked afterward rather than as a basis for setting a target. The a priori benchmark applied was therefore the subject-to-item ratio, for which 10:1 is the common minimum and 20:1 is at the upper end of published recommendations [42]. For a 6-item scale, these ratios correspond to 60 to 120 respondents in the exploratory subsample.

For the CFA, sample size adequacy is commonly evaluated using the ratio of cases to estimated parameters (the N:q rule), for which a ratio of 20:1 is considered ideal and 10:1 an acceptable minimum [37]. A 6-item single-factor specification estimates 12 free parameters (1 loading and 1 residual variance per item), and 1 more for a 2-factor specification, so the rule implies a minimum of 120 to 130 and an ideal of 240 to 260 cases in the confirmatory subsample. Simulation evidence indicates that the requirement for simple confirmatory factor models depends strongly on loading magnitude, with stronger loadings requiring fewer cases [43]; because loading magnitude is itself estimated from the data, that consideration could only be applied after estimation.

In total, if there are at least 180 respondents, both EFA and CFA can be performed. To achieve a more reliable result, the desired sample size is 360.

## Results

### Candidate Items in the New Questionnaire

The 7 candidate items were written so that each targets a distinct, narrowly defined privacy domain drawn from the synthesis mentioned earlier. The domains retain the field’s replicated core (data use, awareness, and control) and extend it to LLM-salient concerns (transparency-for-trust, risk salience, and onward third-party sharing). Table 1 maps each item to its intended domain and to the antecedent constructs in the 6 foundational instruments.

The instrument is deliberately anchored to the most replicated regions of the construct while remaining fit for its new referent. G3 provides a global concern item analogous to overall privacy-concern factors. G2 (awareness) and G1 (data use) instantiate the 2 domains that recur most consistently across the lineage, giving the scale continuity with CFIP, IUIPC, MUIPC, IPC, and the short-form video scale. G5 (control) preserves the control domain, introduced in IUIPC and carried forward in IPC. For LLMs, the domain is especially consequential, because control is exercised through concrete and far-reaching choices: whether user inputs may be used to train future models, and whether conversation history persists across sessions.

The remaining 3 items (G4, G6, and G7) extend these core domains to data practices specific to LLMs. The comparative synthesis of the 6 prior instruments showed that none of them covers these concerns, so each item adds coverage rather than duplicating a domain already represented. G4 reframes awareness as transparency in the service of trust, operationalizing the concern-trust relationship that Hong and Thong [9] established as part of privacy concern’s nomological network and that is acute for opaque, multiparty model providers. G6 (risk salience) captures how frequently the risk is cognitively present, a facet motivated by the demonstrated but often underrecognized LLM risks of memorization and inference [17,18] and by evidence that users disclose to conversational agents under flawed mental models [16]. G7 (onward sharing) sharpens the secondary-use and improper-access concerns of CFIP and IPC into the specific, LLM-relevant question of which third parties receive user data, reflecting the provider ecosystem described earlier. Two items (G2 and G4) intentionally draw on the same awareness or transparency region from complementary angles, notification versus transparency-for-trust, broadening coverage of that domain within a unidimensional design. Framing all 7 items as facets of a single privacy-concern construct, rather than as instruments of one behavioral theory, directly answers the fragmentation in recent LLM survey work, that is, APCO-, uncertainty reduction-, and contextual integrity–based measures each captured a different slice of concern and could not be compared [1-4], whereas the LLM-PCA is designed to be theory-agnostic at the point of measurement.

### Content Validity

All 7 items demonstrated strong content validity on the relevance dimension (Table S1 in Multimedia Appendix 1). Five items (G2 to G6) were endorsed as relevant by all 8 experts (I-CVI=1.000), and the remaining 2 items (G1 and G7) were each endorsed by 7 of the 8 experts (I-CVI=0.875). Every item thus exceeded the 0.78 retention criterion, as well as the more stringent 0.83 threshold recommended for panels of 6 to 8 experts [29]. Chance-corrected agreement was uniformly excellent (modified κ*≥0.871), confirming that the observed endorsement was not attributable to chance [31].

At the scale level, the averaging method yielded an S-CVI/Ave of 0.964, exceeding the 0.90 benchmark for satisfactory content validity. The more conservative UA index was S-CVI/UA=0.714, reflecting the 2 items (G1 and G7) on which a single expert each assigned a rating below 3.

Clarity ratings were also favorable, with item-level indices ranging from 0.750 to 1.000 and an S-CVI/Ave of 0.875. One item (G2) fell below the endorsement threshold on clarity alone (I-CVI=0.750, 2 dissenting experts) while retaining unanimous relevance endorsement. It was retained without modification, although its wording may warrant minor refinement in a future revision.

On the basis of these relevance results, none of the 7 candidate items were removed or reworded, and all were administered to study participants on Qualtrics. The relevance criteria were met by every item, which is the basis for that decision. The one shortfall not acted on was the clarity rating for G2 where I-CVI<0.78. Comprehension of wording is better assessed by cognitive interviewing than by expert rating. Its wording is therefore identified as a known weakness of the current version. It needs to be reassessed using cognitive interviewing in future revisions. The entire questionnaire used in this study is available in Multimedia Appendix 2.

### Participant Characteristics

A total of 603 survey responses were collected. We first removed 11 responses generated during internal pilot testing, leaving 592 responses. Of these, 108 contained no questionnaire data (no answer to any questions) and were excluded, yielding 484 responses. A further 65 respondents reported no awareness of LLMs (37 answered “No” and 28 left the screening item blank) and were routed out of the survey without completing the remaining items; these were excluded, leaving 419 respondents. In the next step, 21 respondents who did not answer any privacy concern survey questions were excluded from the dataset, leaving 398 eligible respondents. Of the 398 eligible respondents, 361 (90.7%) provided complete data on all analysis variables and 37 (9.3%) had partial item nonresponse. Complete and incomplete respondents did not differ significantly on any measured characteristic (Table S2 in Multimedia Appendix 1). Therefore, 37 respondents who omitted 1 or more privacy concern survey items were excluded to retain complete cases only. The resulting analytic sample comprised 361 respondents. This procedure is shown in Figure 2.

**Figure 2.**
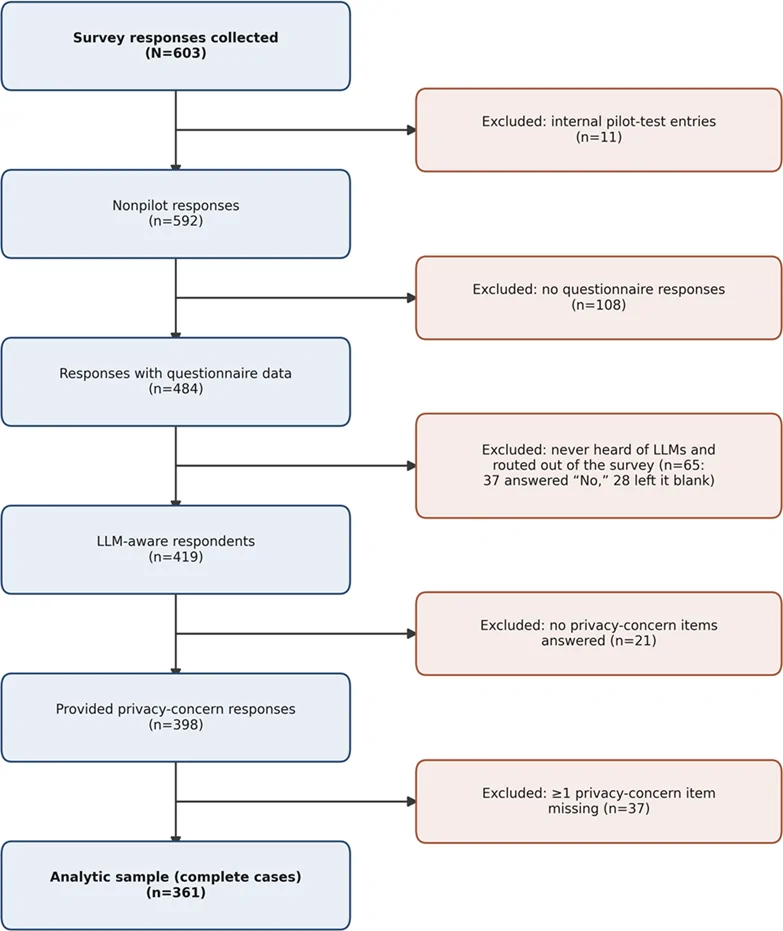
Flowchart of data preprocessing. LLM: large language model.

Of the 361 participants, more than half were younger than 35 years (n=198, 54.8%), most identified as White (n=285, 78.9%), nearly two-thirds were female (n=230, 63.7%), and 88.1% (n=318) held at least a bachelor’s degree. Distributions for all demographic variables are presented in Table 2. Overall, the sample was predominantly White, highly educated, and female, which should be considered when interpreting the generalizability of the factor structure and score distributions.

**Table 2.** Demographics of study participants (N=361).

Demographics	Values, n (%)
Age group (years) (n=342)	
18‐24	141 (39.1)
25‐34	57 (15.8)
35‐44	44 (12.2)
45‐54	44 (12.2)
55‐64	26 (7.2)
≥65	30 (8.3)
Race (n=358)	
Asian or Pacific Islander	31 (8.6)
Black or African American	12 (3.3)
Hispanic or Latino	12 (3.3)
White	285 (78.9)
Mixed race	15 (4.2)
Other	3 (0.8)
Sex (n=359)	
Male	109 (30.2)
Female	230 (63.7)
Nonbinary or prefer not to answer	20 (5.5)
Education level (n=360)	
High school or less	6 (1.7)
Some college or associate degree	34 (9.4)
Bachelor’s degree	117 (32.4)
Master’s degree	102 (28.3)
Professional degree (eg, MD and JD)	24 (6.6)
Doctorate (PhD and EdD)	75 (20.8)
Other	2 (0.6)
Marital status (n=358)	
Single or never married	153 (42.4)
Married or domestic partnership	175 (48.5)
Separated	3 (0.8)
Divorced	18 (5)
Widowed	9 (2.5)
Employment status (n=357)	
Employed for wages (full-time)	242 (67)
Employed for wages (part-time)	23 (6.4)
Self-employed	10 (2.8)
Out of work	9 (2.5)
Homemaker	2 (0.6)
Student	47 (13)
Retired	21 (5.8)
Unable to work	3 (0.8)
Annual income (n=358)	
Less than US $20,000	31 (8.6)
Between US $20,000 and $50,000	86 (23.8)
Between US $50,000 and $80,000	91 (25.2)
Between US $80,000 and $110,000	55 (15.2)
Between US $110,000 and $140,000	30 (8.3)
Between US $140,000 and $180,000	15 (4.2)
Between US $180,000 and $240,000	12 (3.3)
Between US $240,000 and $300,000	7 (1.9)
More than US $300,000	3 (0.8)
Prefer not to answer	28 (7.8)

### Item Descriptive Statistics

Item-level descriptive statistics for the 7 privacy concern items are presented in Table 3, including mean, SD, frequency of participants choosing each option in each question, and anchor type (concern, importance, or frequency). Items were rated on a Likert scale from 1 (extremely important, extremely concerned, or always) to 5 (not at all important, not at all concerned, or never). Therefore, lower values indicate stronger endorsement. Scoring, including the reverse-coding step, is specified in the Methods section. Item means ranged from 1.94 (G5) to 2.51 (G3) on the 1‐5 Likert scale, indicating that, on average, participants endorsed moderate-to-high levels of privacy concern (with lower values indicating stronger endorsement). Item-level SDs were between 0.97 and 1.18. Because item dispersion was narrow and similar across items, a unit-weighted composite and one formed from standardized item scores would differ only slightly in this sample. All items used the full 1‐5 response range. Skewness and kurtosis were calculated as well but not included in the table. Skewness ranged from 0.18 to 0.85 (maximum |skew|=0.85), and kurtosis ranged from −0.94 to 0.04 (maximum |kurtosis|=0.94), all well within conventional bounds (|skew|<2, |kurtosis|<7) for treating items as approximately continuous. Nevertheless, given the 5-point Likert response format, we used polychoric correlations for EFA and DWLS estimation for CFA throughout.

**Table 3.** Descriptive statistics and answer distribution for the 7 privacy concern items (N=361)^a^.

Item	Mean (SD)	1, n (%)	2, n (%)	3, n (%)	4, n (%)	5, n (%)	Anchor type
G1	2.50 (1.14)	91 (25.2)	86 (23.8)	109 (30.2)	63 (17.5)	12 (3.3)	Concern
G2	2.05 (0.97)	122 (33.8)	131 (36.3)	79 (21.9)	25 (6.9)	4 (1.1)	Importance
G3	2.51 (1.18)	98 (27.1)	76 (21.1)	107 (29.6)	66 (18.3)	14 (3.9)	Concern
G4	2.07 (1.00)	125 (34.5)	122 (33.8)	85 (23.5)	22 (6.1)	7 (1.9)	Importance
G5	1.94 (0.99)	151 (41.8)	113 (31.3)	70 (19.4)	22 (6.1)	5 (1.4)	Importance
G6	2.36 (1.08)	89 (24.7)	120 (33.2)	94 (26)	48 (13.3)	10 (2.8)	Frequency
G7	2.37 (1.01)	74 (20.5)	139 (38.5)	94 (26)	48 (13.3)	6 (1.7)	Importance

a Data in columns 1‐5 indicate the frequency of participants choosing one particular option as the answer to each question.

### Instrument Refinement

The questionnaire was initially developed with 7 items, 1 for each privacy-concern domain. Before finalizing the scale, we examined the interitem polychoric correlation matrix and the fit of the 7-item unidimensional model on the confirmatory subsample (n=240). This examination revealed that items G1 (“How concerned are you about the ways in which LLMs use your personal data?”) and G3 (“How much concern do you have for the privacy of your data when using applications or services that use LLMs?”) were nearly collinear, with a polychoric correlation of 0.91. This indicated that both items assess a respondent’s privacy concern with data use, and the strong correlation indicated that they functioned as effectively interchangeable indicators.

To determine which item to remove, we compared 2 reduced 6-item models, one omitting G1 and one omitting G3. Removing G3 produced uniformly good fit (*χ*^2^_9_=13.6; *P*=.14; RMSEA=0.046; SRMR=0.057), whereas removing G1 produced only marginally acceptable fit (*χ*^2^_9_=17.4; *P*=.04; RMSEA=0.062; SRMR=0.065). In the G1-omitted model, the retained item G3 was the weakest indicator (standardized λ=0.74; communality=0.47), whereas in the G3-omitted model, all retained items loaded more strongly. Because removing G3 yielded superior model fit and stronger indicators, we retained G1 and removed G3. The resulting 6-item scale (G1, G2, G4, G5, G6, and G7) was carried forward for all analyses reported below. Removal of an empirically redundant, conceptually overlapping item is a standard step in scale development; retaining both would have overweighted a single domain and degraded model fit without adding measurement information. As reported in the Stability Across Alternative Sample Splits section, this removal decision was reproduced in 5 independent exploratory subsamples without consulting the confirmatory subsample.

### Reliability

Cronbach α for this 6-item questionnaire was 0.90. Because α increases with both the number of items and the average interitem correlation, this value should be interpreted as reflecting high interitem redundancy in a short scale rather than as independent evidence of measurement quality [33]; McDonald ω, computed from the CFA (ω=0.90), is reported below as a complementary estimate.

### EFA

EFA was conducted on the randomly selected EFA subsample (n=121). ML extraction was applied to the polychoric correlation matrix. The polychoric correlations (Table S3 in Multimedia Appendix 1) demonstrated strong but nonredundant interitem association, with off-diagonal correlations ranging from 0.45 (G6 vs G7) to 0.79 (G2 vs G5); removing G3 eliminated the near-collinear pair present in the original 7-item pool. The matrix was well-suited to factor analysis since the overall KMO measure of sampling adequacy was 0.87, in the “good” range, with per-item KMO values from 0.84 (G5) to 0.92 (G7), all above the 0.60 minimum. Bartlett test of sphericity was significant (*χ*^2^_15_=477.4; *P*<.001), confirming that the correlation matrix departed substantially from an identity matrix.

Parallel analysis based on 500 simulated datasets supported a 1-factor solution in the exploratory subsample. The first observed eigenvalue (4.17) substantially exceeded the corresponding 95th-percentile random eigenvalue (1.44), whereas the second observed eigenvalue (0.65) fell below its random benchmark (1.25). Kaiser’s eigenvalue-greater-than-one criterion likewise retained a single factor. The ratio of the first to the second eigenvalue (6.46) further supported essential unidimensionality. The first eigenvalue accounted for 69.6% of total variance, and after ML extraction, the single-factor solution accounted for 63.6% of common variance. Standardized loadings on the retained factor (Table 4) were uniformly substantial in absolute value, ranging from 0.71 (G7) to 0.88 (G5), and all 6 items exceeded the stricter conventional λ≥0.70 threshold for strong indicator quality. The items with the weakest loadings (G6 and G7) may be candidates for rewording or replacement in future work. Item communalities ranged from 0.50 (G7) to 0.78 (G5). The pattern of eigenvalues, the parallel-analysis decision, and the uniformly high single-factor loadings provided convergent evidence for a dominant general factor in the exploratory subsample and supported fitting the planned single-factor CFA model on the holdout subsample.

**Table 4.** Standardized factor loadings from the exploratory factor analysis (EFA; n=121) and from the confirmatory factor analysis (CFA; n=240) for the 6-item large language model privacy concern assessment.

Item	EFA λ^a^	EFA *h*^2^	CFA λ^b^	CFA *R*^2^	Residual^c^
G1	0.77	0.60	0.79^d^	0.62	0.38
G2	0.85	0.72	0.85^d^	0.73	0.27
G4	0.82	0.67	0.83^d^	0.68	0.32
G5	0.88	0.78	0.81^d^	0.65	0.35
G6	0.74	0.55	0.69^d^	0.47	0.53
G7	0.71	0.50	0.69^d^	0.48	0.52

a Maximum likelihood extraction on the polychoric correlation matrix.

b Diagonally weighted least square estimation on the polychoric correlation matrix, with the latent factor variance fixed to 1.0.

c Standardized residual variance.

d *P*<.001.

### CFA

#### Global Fit

The unidimensional 6-item CFA model was fitted to the holdout subsample (n=240) using DWLS estimation. The model exhibited excellent global fit: *χ*^2^_9_=13.6; *P*=.14; CFI=0.996; TLI=0.993; RMSEA=0.046, 90% CI 0.000-0.093; SRMR=0.057. The nonsignificant model chi-square and CFI/TLI values approaching unity indicated that the unidimensional 6-item model reproduced the observed interitem associations closely. The RMSEA point estimate (0.046) fell within the range conventionally considered to reflect close fit (≤0.06), and the test of close fit was clearly nonsignificant (PCLOSE=.50, testing H₀: RMSEA≤0.05). SRMR (0.057) fell well within the range for acceptable fit. GFI and AGFI were strong (GFI=0.99; AGFI=0.98). All 6 standardized factor loadings were positive, substantial, and statistically significant (*P*<.001), with values of 0.79 (G1), 0.85 (G2), 0.83 (G4), 0.81 (G5), 0.69 (G6), and 0.69 (G7). Standardized loadings, *R*^2^ values, and standardized residual variances are presented in Table 4. In total, 4 of the 6 items exceeded the conventional λ≥0.70 threshold for strong indicator quality, and the remaining 2 (G6 and G7, both λ=0.69) fell just below it while remaining well above the minimum acceptable value of 0.50. Item *R*^2^ values ranged from 0.47 (G6) to 0.73 (G2), indicating that the latent privacy concern factor accounted for between 47% and 73% of the variance in each item. Figure 3 shows the path diagram used in the CFA for the unidimensional model with 6 items.

**Figure 3.**
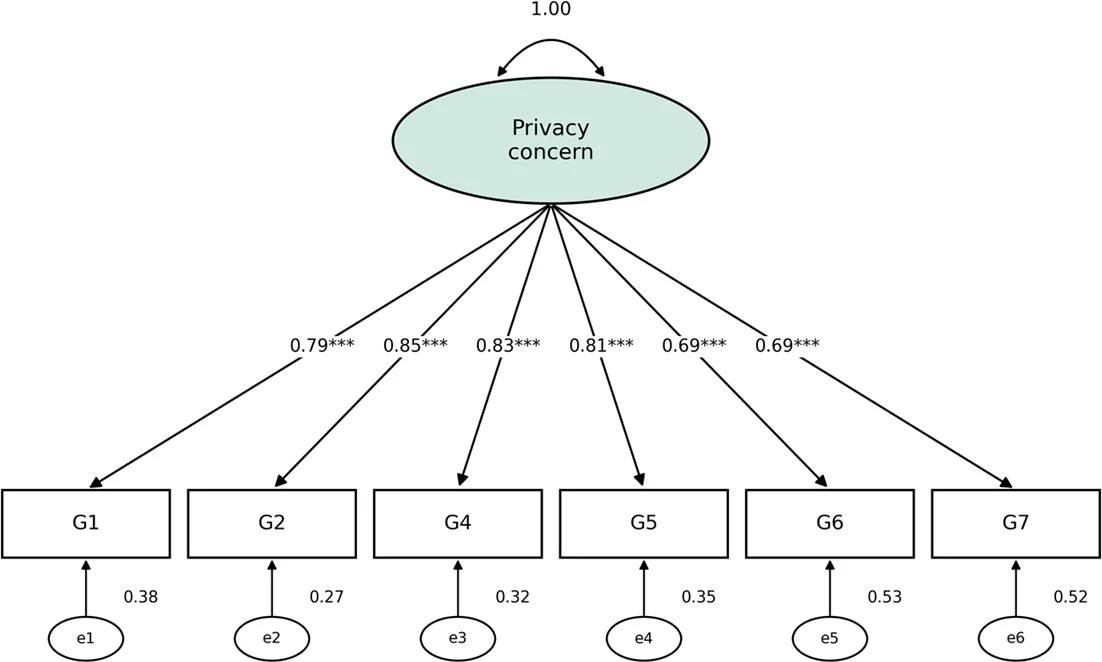
Confirmatory factor analysis of the 6-item large language model-privacy concern assessment questionnaire. Standardized factor loadings are shown on the paths from the latent factor to each indicator; standardized residual variances are shown next to each error term (e1-e6). The variance of privacy concern was fixed at 1.00 for identification of the standardized solution. All loadings significant at ***P<.001.

Composite reliability was excellent (ω=0.90) above the conventional 0.80 threshold for very good internal consistency, and the AVE was 0.61, exceeding the 0.50 Fornell-Larcker convergent-validity threshold. Taken together, these results support the internal consistency and convergent validity of the refined LLM-PCA at the level of the full 6-item set. The dimensional structure is addressed in the Comparison of 1-Factor and 2-Factor Models section.

#### Local Fit

Because strong global fit does not guarantee adequate fit at the level of individual item pairs, we examined the residual correlation matrix (Table S4 in Multimedia Appendix 1). Residuals are the differences between observed polychoric correlations and model-implied correlations. Most residuals were small, but 4 of the 15 off-diagonal residual correlations exceeded the 0.10 threshold: G1-G6 (residual=0.21), G2-G5 (residual=0.18), G4-G5 (residual=0.15), and G2-G4 (residual=0.14). The root mean square residual (computed on the polychoric matrix) across all off-diagonal elements was 0.098. In total, 3 of the 4 elevated residuals involved the transparency- and control-oriented items G2 (being informed about data use), G4 (transparency supporting trust), and G5 (control over data use), indicating that these conceptually adjacent items share covariance beyond what the general privacy-concern factor accounts for. The remaining elevated residual, between G1 (data use) and G6 (frequency of thinking about privacy risks), reflects a smaller secondary association. These residuals indicate that although a strictly unidimensional model provides excellent global fit and a robust SRMR (computed on the Pearson covariance), it does not fully reproduce the local covariance among the transparency- and control-related items.

### Comparison of 1-Factor and 2-Factor Models

Because the residual analysis indicated localized covariance among the transparency- and control-related items, we compared the unidimensional model (M1) with a 2-factor alternative (M2) on the same CFA subsample (n=240). The 2-factor model assigned the transparency- and control-oriented items to 1 factor (G2, G4, and G5) and the remaining items to a second factor (G1, G6, and G7), with the 2 factors allowed to correlate. Both models were estimated with DWLS on the polychoric correlation matrix. Fit statistics for the 2 models are presented in Table 5.

**Table 5.** Comparison of 1-factor and 2-factor confirmatory models (confirmatory factor analysis subsample, n=240).

Model	*χ*^2^ (*df*)	*P* value	CFI^a^	TLI^b^	RMSEA^c^ (90% CI)	SRMR^d^	AIC^e^	BIC^f^
One-factor (M1)	13.6 (9)	.14	0.996	0.993	0.046 (0.000-0.093)	0.057	23.21	64.97
Two-factor (M2)	4.7 (8)	.79	1.00	1.00	0.000 (0.000-0.050)	0.032	25.74	70.99

a CFI: comparative fit index.

b TLI: Tucker-Lewis index.

c RMSEA: root mean square error of approximation.

d SRMR: standardized root mean square residual.

e AIC: Akaike information criterion.

f BIC: Bayesian information criterion.

The 2-factor model fits better than the 1-factor model on every index examined: (*χ*^2^_8_=4.7; *P*=.79 vs *χ*^2^_9_=13.6; *P*=.14; CFI and TLI of 1.00 vs 0.996 and 0.993; RMSEA=0.000, 90% CI 0.000-0.050 vs 0.046, 90% CI 0.000-0.093; SRMR=0.032 vs 0.057). The nested chi-square difference test was significant (Δ*χ*^2^_1_=8.9; *P*=.003), consistent with the localized residual covariance identified earlier. Therefore, the 2-factor specification is the better-fitting model on these data, and the improvement is not marginal. Table 5 also reports the Akaike information criterion and the Bayesian information criterion, both of which favor the 1-factor model. These criteria are likelihood-based, and because the models were estimated by DWLS rather than by ML, the values are derived from the fit function rather than a true likelihood and are not interpretable on their usual scale. They are reported for completeness, and the nested chi-square difference test was used for the model comparison.

We therefore retain the 2-factor solution as the measurement model for the LLM-PCA. The first factor is defined by the transparency- and control-related items (G2, being informed about data use; G4, transparency supporting trust; and G5, control over data use) and is labeled transparency and control. The second is defined by the remaining items (G1, concern about data use; G6, frequency of thinking about privacy risks; and G7, onward third-party sharing) and is labeled data use and risk salience. Standardized loadings in the 2-factor solution ranged from 0.85 to 0.90 on the first factor and from 0.72 to 0.85 on the second.

One property of this solution affects how it should be used. The 2 latent factors correlated at *r*=0.83; therefore, they share approximately 69% of their variance. The evidence on discriminant validity is mixed. The HTMT was 0.85 (<0.90), which indicates that these 2 factors are empirically distinct. The Fornell-Larcker criterion was met for transparency and control, whose AVE (0.77) exceeds the squared factor correlation (0.69), but not for data use and risk salience, whose AVE (0.59) falls below it. In other words, the 2 factors are separable but not fully distinct. The subscale reliability was acceptable (transparency and control: α=0.91; ω=0.91; data use and risk salience: α=0.81; ω=0.81). For these reasons, the 6-item total score remains the recommended primary metric for applied use, and the subscale scores, each a unit-weighted mean of its 3 items, should be reported as secondary, facet-level information.

### Stability Across Alternative Sample Splits

To verify that the reported results were not an artifact of the particular random partition, the entire split-sample procedure (item refinement, EFA, and CFA) was repeated under 5 different random partitions, holding the one-third exploratory, two-thirds confirmatory allocation fixed. The findings were stable across all 5 splits. The near-collinearity of the 2 general-concern items exceeded the 0.85 threshold in every exploratory subsample, and the reduced-model comparison, conducted on the exploratory subsample without consulting the confirmatory subsample, indicated removal of G3 in all 5 cases. Parallel analysis retained a single factor in every exploratory subsample (first-to-second eigenvalue ratio=4.3‐7.6; KMO=0.82-0.87; minimum exploratory loading=0.57-0.71). The confirmatory model showed acceptable-to-excellent global fit on every holdout subsample (*χ*^2^_9_=7.7‐15.8; all *P*≥.07; CFI≥0.993; RMSEA≤0.056; SRMR≤0.064; PCLOSE≥.36), with composite reliability ω=0.90-0.91 and AVE=0.59-0.64. The localized residual strain among the transparency- and control-related items replicated in every split (4-5 residual correlations exceeding 0.10 per split), and G6 and G7 were the weakest indicators in each confirmatory solution (standardized λ=0.67-0.75), consistent with the pattern reported earlier.

## Discussion

### Principal Findings

This study developed and validated a concise 6-item instrument, the LLM-PCA, for assessing users’ privacy concerns regarding LLM-based systems. There are 4 major findings in this study. First, the 7 candidate domains could be reduced to 6 items without loss of content coverage, because 2 general concern items proved to be near-interchangeable indicators. Therefore, one of them was removed from the instrument. Second, the final instrument demonstrated high internal consistency and a clearly dominant general factor in the exploratory subsample. Third, a confirmatory model fitted to an independent holdout subsample reproduced the observed interitem associations closely, and the whole procedure replicated across 5 alternative random partitions of the sample, indicating that the findings are not an artifact of one split. Fourth, the covariance among the transparency- and control-related items is not fully explained by a single general factor. A 2-factor model fit the data better than a 1-factor model and was retained as the measurement model, separating a transparency and control facet from a data use and risk salience facet. The 2 factors met one of the 2 conventional discriminant validity criteria but not the other, so the instrument is offered as a brief 2-facet measure whose total score remains the recommended primary metric for privacy concern with LLM applications, with facet scores providing secondary detail. To our knowledge, it is among the first peer-reviewed instruments developed specifically for this referent, addressing a gap in the growing body of survey-based research in this area.

### Comparison With Prior Work

As described in the Introduction section, prior LLM privacy surveys each used a bespoke instrument built for a different theoretical framework and a different outcome; therefore, the concern levels they report cannot be placed on a common metric with LLM-PCA scores. The comparison drawn here is one of structure and measurement properties rather than of score levels. We do not claim that the instrument derives from, or validates, any single theoretical framework; establishing its theoretical grounding and construct validity will require further work. Table S5 in Multimedia Appendix 1 summarizes the comparison of the LLM-PCA with prior privacy concern instruments and recent LLM privacy studies.

The factor structure observed here should be read against prior work. Earlier general privacy concern scales, such as those developed for organizational, e-commerce, and short-form video platform contexts, have often yielded multifactor solutions capturing dimensions such as collection, unauthorized access, secondary use, and awareness [6,7,10,11]. The present results are consistent with that literature. A 2-factor solution separating transparency and control items from data use and risk salience items fit better than a single-factor solution, and those 2 facets correspond closely to the control and awareness dimensions identified in earlier instruments [7,9]. What the present design cannot do is resolve those facets into well-identified factors, because each domain is represented by a single item and each candidate factor is therefore defined by only 3 indicators. Reporting a single composite score is a decision about parsimony and applied usability at this scale length, not a finding that users hold an undifferentiated view of privacy. Whether privacy concern with LLMs is best represented by 1 general factor or by 2 correlated facets is a question for a longer instrument with multiple indicators per domain. The possibility that an early stage of public understanding contributes to the high correlation between the 2 facets is consistent with findings from Choi et al [1], who found that 66% of their sample had limited knowledge of available privacy protection options in LLMs, and from Liu et al [3], who observed a systematic underestimation of health data risk and a gap between users’ stated privacy concerns and their actual expectations. As public understanding of LLM data practices matures, multidimensional solutions may emerge in future validation studies involving more experienced user populations.

### Implications

The validated instrument offers practical utility for both researchers and practitioners. For researchers, the availability of a standardized 6-item scale makes it feasible to incorporate privacy concern assessment as a routine component of LLM user studies without imposing significant respondent burden. Its single composite score, with demonstrated reliability, enables meaningful cross-study comparisons, a capability that has been largely absent from the existing literature. For practitioners and AI application developers, the scale provides a concise, validated tool for benchmarking user privacy concern levels before and after system design changes, privacy policy updates, or new feature deployments, supporting data-driven evaluation of user-facing privacy interventions.

The scale’s focus on LLM-specific concerns, including data use practices, transparency, user control, and organizational data sharing, makes it particularly relevant for the growing number of commercial and enterprise LLM deployments in health care, education, legal services, and other sensitive domains. In health care contexts, where prior work has shown that privacy concerns can shape users’ engagement with AI-assisted consultation [1-3], this instrument could support both predeployment risk assessment and ongoing postdeployment monitoring of user trust. For example, health care organizations deploying LLM-based tools such as clinical decision support systems, patient-facing chatbots, or electronic health record–integrated assistants could administer the scale prior to deployment to establish a baseline of user privacy concern, and again following deployment to detect shifts in trust associated with system changes or privacy policy updates. Because the instrument yields a single interpretable score, it is practical for routine use in clinical and operational settings without requiring psychometric expertise. As a preliminary interpretive aid pending formal norming, and after reverse-coding so that higher scores denote greater concern, mean item scores in the upper part of the scale (roughly ≥4 of 5) may be read as indicating high privacy concern, scores near the midpoint as moderate concern, and scores in the lower part (roughly ≤2) as low concern. These provisional bands are illustrative only and should be calibrated against representative normative samples before being used to guide decisions.

### Limitations and Future Work

Several limitations should be considered when interpreting findings reported in this study. First, the sample skewed toward younger, White, highly educated, and predominantly female participants, characteristics that likely reflect the early-adopter demographic of LLM technology. Because privacy attitudes toward AI systems may vary with age, educational background, digital literacy, cultural background, and prior technology exposure, the generalizability of both the scale’s factor structure and the observed levels of concern to more diverse populations cannot be assumed. In particular, the overrepresentation of highly educated individuals may mean that the scale’s factor structure reflects a relatively privacy-literate population. Replication studies with demographically diverse and community-based samples are needed to evaluate measurement invariance across groups. Because the sex item combined nonbinary respondents with those who preferred not to answer into one category, the response set was less inclusive than current best practice and did not allow sex-diverse participants to be identified separately; future versions should offer distinct nonbinary and self-describe options in addition to a prefer-not-to-answer choice. Because most respondents identified as White and held at least a bachelor degree, the score distributions reported here should not be treated as normative values for the US adult population, and the provisional interpretive bands suggested in the Implications section should not be applied to more diverse populations until they have been recalibrated. Differential item functioning across education, age, and racial or ethnic groups is untested, so the items may not carry the same meaning among respondents less familiar with LLM data practices, which would affect the factor structure as well as the observed levels of concern.

Second, the questionnaire was validated as a measure of general privacy concerns with LLMs rather than concerns tied to a specific application or use context. While this generality is a strength for cross-study comparability, it means that the instrument’s criterion validity, its ability to predict behaviorally relevant outcomes such as disclosure willingness, trust, or system abandonment, remains to be established. Future research should administer the scale alongside behavioral outcome measures and within the context of specific LLM applications, such as AI-assisted clinical decision support or consumer-facing chatbots, to evaluate its predictive utility across different use cases. More specifically, convergent and criterion validity could be established through several complementary approaches. Concurrent validity could be assessed by administering the LLM-PCA alongside established multi-item privacy-concern instruments, such as the CFIP scale, the IUIPC scale, the MUIPC scale, and Hong and Thong’s integrated model, within the same sample; moderate-to-strong positive correlations would support convergent validity, whereas weaker associations with conceptually adjacent but distinct constructs, such as general institutional trust, technology acceptance, or privacy self-efficacy, would provide discriminant evidence and help situate the instrument within its nomological network. Criterion validity could then be evaluated against behaviorally relevant outcomes assessed by self-report or, preferably, by observation: the willingness to disclose personal and sensitive (eg, health) information to an LLM, the amount and type of information actually disclosed in logged interactions, the adoption and continued use versus abandonment of an LLM application, and the uptake of privacy-protective options such as opting out of model training, deleting conversation history, or anonymizing inputs. Known-groups comparisons would provide a further line of evidence, with higher concern scores expected among respondents who have previously experienced a privacy breach, who routinely handle sensitive data, or who work in highly regulated professions, such as health care. Because concurrent designs can establish only association, prospective designs in which baseline LLM-PCA scores are used to predict subsequently observed disclosure or adoption behavior would be needed to demonstrate predictive validity, and vignette-based experiments that manipulate contextual factors, for example, the recipient of the data, the purpose of processing, or the presence of consent, would test whether scores respond to theoretically meaningful changes in the information flow.

Third, the study relied on convenience sampling via a single university and its affiliated recruitment platforms. This limits the representativeness of the sample with respect to occupation, geographic region, and general population awareness of LLMs. Future validation studies should target broader, population-based samples.

Fourth, although both subsamples met the a priori benchmarks set out in the Methods section, the split-sample cross-validation design produced an EFA subsample of n=121 and a CFA subsample of n=240. These subsample sizes are modest in absolute terms but adequate for this initial validation: the EFA subsample satisfied the conditions under which small-sample factor recovery is reliable (high communalities and a strongly overdetermined single factor, with KMO=0.87), and the CFA subsample was at or close to the ideal 20:1 ratio of cases to estimated parameters, depending on which specification is counted. The evaluation is post hoc: confirming after the fact that the favorable condition held is weaker evidence than a prospective power analysis would have provided. Nonetheless, the modest absolute sizes may have reduced the precision of factor-loading estimates and the power of fit indices to detect localized misspecification. The 1- versus 2-factor comparison is the analysis most affected: with 3 indicators per factor at this subsample size, it can show that the 2-factor model fits better, but not how the 2 facets behave in relation to external criteria. Replication of the factor structure in independent and larger samples, ideally drawn from distinct subgroups to permit measurement-invariance testing, remains an important next step before the scale is treated as a fully validated instrument.

Fifth, the instrument uses heterogeneous response anchors across items (importance, concern, and frequency) on a common 1‐5 scale. While this is appropriate for the conceptual content of each item, it assumes that respondents treat position 3 on each anchor type as functionally equivalent. Future work should evaluate whether converting all items to a single anchor type (eg, agreement with statements) yields comparable psychometric properties and potentially simplifies scoring. In addition, composite scores are formed by unit-weighted summation of raw responses without prior standardization, which assumes that the 5 ordinal positions are metrically equivalent across the 3 anchor types. That assumption was not tested here, and the reasons for retaining unit weighting are set out in the Methods section. Future work should compare unit-weighted with standardized scoring in the same sample and report whether substantive conclusions differ.

Sixth, the items were pretested only within the study team and did not undergo cognitive interviewing with lay respondents before fielding. Several items use relatively abstract phrasing and a comparatively high reading level, and the awareness screening item may not have been interpreted uniformly by lay respondents. Because these issues bear directly on content validity, the instrument should be regarded as preliminary, and future work should incorporate cognitive pretesting and readability assessment in community samples. Relatedly, the expert review cannot substitute for the pretesting that was not done. The panel comprised 8 reviewers identified through research team recommendation, which meets the recommended panel size but is small and not independently constituted, and it assessed clarity by inspection rather than by observing how respondents interpreted the items. The obtained clarity index for G2 fell below the 0.78 item-level criterion because 2 of the 8 reviewers rated below the endorsement threshold despite unanimous endorsement of its relevance. It was retained unchanged, so the wording the expert panel flagged has not been tested on lay respondents. This is a known weakness of the current version.

Seventh, temporal stability of the instrument was not assessed. Because internal consistency cannot establish reliability for single-item domains, test-retest reliability over an appropriate interval is an important form of evidence that remains to be collected in a future study.

Eighth, all data were self-reported, so common method bias cannot be ruled out and associations involving the construct may be inflated relative to multimethod designs [44].

In addition, the items with the weakest loadings (G6 and G7 0.74 and 0.71, respectively) may be candidates for rewording or replacement in future work. Because each domain is represented by a single item, future multi-item per domain development could add parallel indicators for the risk-salience (G6) and onward third-party-sharing (G7) domains, or revise their current wording, to improve their measurement precision. More generally, the one-item-per-domain design carries costs. It yields no within-domain reliability estimate, so no domain score can be corrected for measurement error. It makes each domain maximally sensitive to the wording of a single stem, which is precisely the vulnerability the interpretation literature documents [12,13]. It precludes tests of within-domain measurement invariance across groups. It also limits how well any latent facet can be identified, as the 1- versus 2-factor comparison illustrates. These costs were accepted in exchange for content breadth and brevity, and they are the main reasons the instrument is presented as a brief screening and benchmarking measure rather than as a substitute for a full multidimensional battery.

### Conclusions

This study addressed a recognized gap in the LLM privacy research literature by developing and preliminarily validating a concise 6-item instrument for assessing user privacy concerns with LLM-based systems. The scale demonstrated high internal consistency, and a comparison of 1- and 2-factor specifications supported a 2-factor structure comprising a transparency and control facet and a data use and risk salience facet. Because the 2 facets are highly correlated and only partly distinct, the 6-item total score is the recommended primary metric, with facet scores providing secondary detail. Unlike prior instruments in this domain, which were developed within specific theoretical frameworks for narrow research purposes, this LLM-PCA instrument is designed for broad applicability, enabling cross-study comparisons and practical privacy assessments by researchers and AI application developers alike. Future work should prioritize replication in demographically diverse samples, evaluation of criterion validity against behavioral outcomes, and administration within specific LLM application contexts to further establish the instrument’s utility and boundary conditions.

## Supplementary material

10.2196/102910 Multimedia Appendix 1 Tables S1-S5 reporting content validity, nonresponse, correlation, and model-fit results supporting the development and validation of the large language model privacy concern assessment.

10.2196/102910 Multimedia Appendix 2 A copy of the complete questionnaire.

## Abbreviations

AGFI: adjusted goodness-of-fit index
APCO: antecedents, privacy concerns, and outcomes
AVE: average variance extracted
CFA: confirmatory factor analysis
CFI: comparative fit index
CFIP: Concern for Information Privacy
CVI: content validity index
DWLS: diagonally weighted least squares
EFA: exploratory factor analysis
GFI: goodness-of-fit index
HTMT: heterotrait-monotrait ratio of correlations
I-CVI: item-level content validity index
IPC: Internet Privacy Concerns
IUIPC: Internet Users’ Information Privacy Concerns
KMO: Kaiser-Meyer-Olkin
LLM: large language model
LLM-PCA: large language model privacy concern assessment
ML: maximum likelihood
MUIPC: Mobile Users’ Information Privacy Concerns
PCLOSE: *P* value for the test of close fit
RMSEA: root mean square error of approximation
S-CVI: scale-level content validity index
SRMR: standardized root mean square residual
TLI: Tucker-Lewis index
UA: universal-agreement
