# Supplementary material for: Development and Validation of the Large Language Model Privacy Concern Assessment Questionnaire: Psychometric Evaluation Study on a Brief Instrument for Assessing User Privacy Concerns With Large Language Model Applications
Source: JMIR Hum Factors. 2026 Oct 21;13:e102910. doi: 10.2196/102910 (PMC13686613; doi:10.2196/102910)
Supplement: Supplementary file 1 [file humanfactors-v13-e102910-s002.pdf]

Questionnaire Used in LLM-PCA Study

Demographics

1. What is your age?
    - 18-24 years old
    - 25-34 years old
    - 35-44 years old
    - 45-54 years old
    - 55-64 years old
    - 65 years or older

2. Please specify your race.
    - White
    - Hispanic or Latino
    - Black or African American
    - Asian/Pacific Islander
    - Mixed Race
    - Other (please specify)

3. What is your gender identity?
    - Male
    - Female
    - Neither/prefer not to answer

4. What is your highest level of education?
    - High school or less
    - Some college or associate degree
    - Bachelor’s degree
    - Master’s degree
    - Professional degree (e.g., MD, JD)
    - Doctoral (PhD, EdD, etc.)
    - Other (please specify)

5. What is your marital status?
    - Single/never married
    - Married or domestic partnership
    - Separated
    - Divorced
    - Widowed

6. What is your current employment status?
    - Employed for wages (full-time)
    - Employed for wages (part-time)
    - Self-employed
    - Out of work
    - Homemaker
    - Student
    - Retired
    - Unable to work

7. What is your annual income?
    - Less than $20,000
    - Between $20,000-$50,000
    - Between $50,000-$80,000
    - Between $80,000-$110,000
    - Between $110,000-$140,000
    - Between $140,000-$180,000
    - Between $180,000-$240,000
    - Between $240,000-$300,000
    - More than $300,000
    - Prefer not to answer

Screening question

8. Have you heard of large language models (LLMs*) before? LLM stands for large language model, which is a type of artificial intelligence (AI) program that can generate and understand texts and perform other tasks. For instance, ChatGPT is based on an LLM.
    - Yes
    - No

Note: If participants answered No or did not provide a response to this screening question, they would not be allowed to answer the questions below.

LLM-PCA Items

9. How concerned are you about the ways in which LLMs use your personal data?
    - Extremely concerned (1)
    - Very concerned (2)
    - Moderately concerned (3)

- Slightly concerned (4)
- Not at all concerned (5)

10. How important is it for you to be informed about how LLMs use your data?
    - Extremely important (1)
    - Very important (2)
    - Moderately important (3)
    - Slightly important (4)
    - Not at all important (5)

11. How much concern do you have for the privacy of your data when using applications or services that utilize LLMs?
    - Extremely concerned (1)
    - Very concerned (2)
    - Moderately concerned (3)
    - Slightly concerned (4)
    - Not at all concerned (5)

12. How important is transparency about data usage to your trust in LLM-based applications?
    - Extremely important (1)
    - Very important (2)
    - Moderately important (3)
    - Slightly important (4)
    - Not at all important (5)

13. How important is it for you to have control over how LLMs use your data?
    - Extremely important (1)
    - Very important (2)
    - Moderately important (3)
    - Slightly important (4)
    - Not at all important (5)

14. How often do you think about the potential privacy risks when using LLM-based applications?
    - Always (1)
    - Often (2)
    - Sometimes (3)
    - Rarely (4)
    - Never (5)

15. How important is it for you to know about the specific companies or organizations with whom your data is shared?
    - Extremely important (1)
    - Very important (2)
    - Moderately important (3)
    - Slightly important (4)
    - Not at all important (5)
