# Supplementary material for: Development and Validation of the Large Language Model Privacy Concern Assessment Questionnaire: Psychometric Evaluation Study on a Brief Instrument for Assessing User Privacy Concerns With Large Language Model Applications
Source: JMIR Hum Factors. 2026 Oct 21;13:e102910. doi: 10.2196/102910 (PMC13686613; doi:10.2196/102910)
Supplement: Supplementary file 2 [file humanfactors-v13-e102910-s001.docx]

**Table S1**. Item-level and scale-level content validity (relevance ratings; N = 8 experts).

| **Item** | **Experts endorsing (n)** | **I-CVI** | **Modified κ*** | **Interpretation** |
| --- | --- | --- | --- | --- |
| G1 | 7/8 | .875 | .871 | Excellent |
| G2 | 8/8 | 1.000 | 1.000 | Excellent |
| G3 | 8/8 | 1.000 | 1.000 | Excellent |
| G4 | 8/8 | 1.000 | 1.000 | Excellent |
| G5 | 8/8 | 1.000 | 1.000 | Excellent |
| G6 | 8/8 | 1.000 | 1.000 | Excellent |
| G7 | 7/8 | .875 | .871 | Excellent |
| Scale-level CVI, averaging method (S-CVI/Ave) | | .964 | | |
| Scale-level CVI, universal agreement (S-CVI/UA) | | .714 | | |

**Table S2.** Comparison of respondents retained for complete-case analysis (n = 361) with those excluded owing to partial item nonresponse (n = 37). All variables were tested with the Pearson chi-square test of independence, with Cramer V as the effect size. Where expected cell frequencies fell below 5, Monte Carlo P values based on 10,000 replicates, conditioning on both margins, were reported alongside the chi-square statistics. A two-sided α of .05 was applied.

| **Characteristic** | **Retained** **(n = 361)** **n (%)** | **Excluded** **(n = 37)** **n (%)** | **Statistic** | **Effect size**  **(Cramer V)** | **P value** |
| --- | --- | --- | --- | --- | --- |
| **Age group** |  |  | χ²(5) = 5.31 | .119 | .381 |
| 18-24 years old | 141 (39.1) | 19 (51.4) |  |  |  |
| 25-34 years old | 57 (15.8) | 7 (18.9) |  |  |  |
| 35-44 years old | 44 (12.2) | 4 (10.8) |  |  |  |
| 45-54 years old | 44 (12.2) | 2 (5.4) |  |  |  |
| 55-64 years old | 26 (7.2) | 1 (2.7) |  |  |  |
| 65 years or older | 30 (8.3) | 1 (2.7) |  |  |  |
| **Race** |  |  | χ²(5) = 9.33 | .154 | .103 |
| White | 285 (78.9) | 22 (59.5) |  |  |  |
| Hispanic or Latino | 12 (3.3) | 2 (5.4) |  |  |  |
| Black or African American | 12 (3.3) | 3 (8.1) |  |  |  |
| Asian / Pacific Islander | 31 (8.6) | 7 (18.9) |  |  |  |
| Mixed Race | 15 (4.2) | 1 (2.7) |  |  |  |
| Other | 3 (0.8) | 1 (2.7) |  |  |  |
| **Gender (n = 396)** |  |  | χ²(2) = 0.40 | .032 | .878 |
| Male | 109 (30.2) | 11 (29.7) |  |  |  |
| Female | 230 (63.7) | 23 (62.2) |  |  |  |
| Non-binary or prefer not to answer | 20 (5.5) | 3 (8.1) |  |  |  |
| **Education level (n = 396)** |  |  | χ²(6) = 5.87 | .122 | .399 |
| High school or less | 6 (1.7) | 1 (2.7) |  |  |  |
| Some college or associate degree | 34 (9.4) | 2 (5.4) |  |  |  |
| Bachelor's degree | 117 (32.4) | 11 (29.7) |  |  |  |
| Master’s degree | 102 (28.3) | 14 (37.8) |  |  |  |
| Professional degree (e.g., MD, JD) | 24 (6.6) | 3 (8.1) |  |  |  |
| Doctorate (PhD, EdD) | 75 (20.8) | 4 (10.8) |  |  |  |
| **Marital status (n = 394)** |  |  | χ²(4) = 1.76 | .067 | .833 |
| Single/never married | 153 (42.4) | 19 (51.4) |  |  |  |
| Married or domestic partnership | 175 (48.5) | 15 (40.5) |  |  |  |
| Separated | 3 (0.8) | 0 (0.0) |  |  |  |
| Divorced | 18 (5.0) | 1 (2.7) |  |  |  |
| Widowed | 9 (2.5) | 1 (2.7) |  |  |  |
| **Employment status** |  |  | χ²(7) = 4.29 | .105 | .721 |
| Employed for wages (full-time) | 242 (67.0) | 26 (70.3) |  |  |  |
| Employed for wages (part-time) | 23 (6.4) | 4 (10.8) |  |  |  |
| Self-employed | 10 (2.8) | 0 (0.0) |  |  |  |
| Out of work | 9 (2.5) | 0 (0.0) |  |  |  |
| Homemaker | 2 (0.6) | 0 (0.0) |  |  |  |
| Student | 47 (13.0) | 4 (10.8) |  |  |  |
| Retired | 21 (5.8) | 1 (2.7) |  |  |  |
| Unable to work | 3 (0.8) | 0 (0.0) |  |  |  |
| **Annual income** |  |  | χ²(9) = 5.84 | .122 | .755 |
| Less than $20,000 | 31 (8.6) | 4 (10.8) |  |  |  |
| Between $20,000-$50,000 | 86 (23.8) | 9 (24.3) |  |  |  |
| Between $50,000-$80,000 | 91 (25.2) | 9 (24.3) |  |  |  |
| Between $80,000-$110,000 | 55 (15.2) | 8 (21.6) |  |  |  |
| Between $110,000-$140,000 | 30 (8.3) | 0 (0.0) |  |  |  |
| Between $140,000-$180,000 | 15 (4.2) | 1 (2.7) |  |  |  |
| Between $180,000-$240,000 | 12 (3.3) | 2 (5.4) |  |  |  |
| Between $240,000-$300,000 | 7 (1.9) | 0 (0.0) |  |  |  |
| More than $300,000 | 3 (0.8) | 0 (0.0) |  |  |  |
| Prefer not to answer | 28 (7.8) | 3 (8.1) |  |  |  |

Notes. Because the excluded group was small (n = 37) and responses were spread across many categories, these comparisons had limited statistical power, effect sizes were therefore examined alongside the P values and nonsignificant results were interpreted as consistent with, but not confirmation of, ignorable missingness. Responses to the privacy concern items were not compared between the two groups because of high percentage of missingness (e.g., G2, G4, G5, G6 were each missing for more than 30 out of 37 excluded respondents), which was exactly why those cases were excluded from further analysis.

**Table S3.** Polychoric correlations among the six items (n = 121).

|  | **G1** | **G2** | **G4** | **G5** | **G6** | **G7** |
| --- | --- | --- | --- | --- | --- | --- |
| **G1** | 1.00 | .62 | .66 | .63 | .74 | .53 |
| **G2** | .62 | 1.00 | .64 | .79 | .61 | .65 |
| **G4** | .66 | .64 | 1.00 | .74 | .63 | .56 |
| **G5** | .63 | .79 | .74 | 1.00 | .60 | .64 |
| **G6** | .74 | .61 | .63 | .60 | 1.00 | .45 |
| **G7** | .53 | .65 | .56 | .64 | .45 | 1.00 |

**Table S4.** Residual correlation matrix for the six-item Confirmatory Factor Model (n = 240).

|  | **G1** | **G2** | **G4** | **G5** | **G6** | **G7** |
| --- | --- | --- | --- | --- | --- | --- |
| **G1** | — | .01 | .03 | .03 | .21 | .07 |
| **G2** | .01 | — | .14 | .18 | -.03 | .09 |
| **G4** | .03 | .14 | — | .15 | .05 | .02 |
| **G5** | .03 | .18 | .15 | — | -.02 | .05 |
| **G6** | .21 | -.03 | .05 | -.02 | — | .06 |
| **G7** | .07 | .09 | .02 | .05 | .06 | — |

**Table S5.** Comparison of the LLM-PCA with prior privacy concern instruments and recent LLM privacy studies. Study-specific instruments were not designed as standalone and reusable scales.

| **Instrument / study** | **Theoretical framework** | **# items** | **Construct measured** | **Validation status** | **Intended use** |
| --- | --- | --- | --- | --- | --- |
| CFIP [1] | Social-contract / normative view of organizational practices | 15 | Concern for information privacy: collection, unauthorized secondary use, improper access, errors | Extensively validated; second-order structure confirmed [2] | Organizational / direct-marketing data practices |
| IUIPC [3] | Social contract theory | 10 | Internet users' privacy concern: collection, control, awareness | Validated within a trust–risk causal model | General online / e-commerce contexts |
| MUIPC [4] | Communication Privacy Management | 9 | Mobile users' privacy concern: perceived surveillance, perceived intrusion, secondary use | Validated in mobile-app context | Mobile applications |
| Choi et al. (2025) [5] | APCO | Study-specific | Privacy concern as a mediator; outcome = willingness to disclose health information to LLMs | Regression / mediation model; not offered as a validated scale | Explaining disclosure willingness in health LLM use |
| Liu, Zou & Lin (2025) [6] | Uncertainty Reduction Theory | Study-specific | Privacy concern as a predictor of AI-chatbot use | Survey-based behavioral-prediction model | Predicting AI-chatbot use in healthcare |
| Liu, Hu, et al. (2025) [7] | Contextual integrity | Study-specific | Privacy awareness/expectations; attitude–behavior gap | Deployment + user survey | Understanding users' expectations toward LLM health consultation |
| Tran et al. (2025) [8] | Contextual integrity | Vignette factors | Appropriateness of information flows / privacy norms | Survey experiment; mixed models | Identifying which factors (consent, de-identification) shape appropriateness |
| **LLM-PCA (this study)** | Theory-agnostic; eclectically informed by prior frameworks | **6** | General user privacy concern with LLM applications | Content validity, EFA + CFA, split-sample validation | Brief, standardized, repeatable measurement of LLM privacy concern for research and practice |

**References**

1. Smith HJ, Milberg SJ, Burke SJ. Information Privacy: Measuring Individuals’ Concerns About Organizational Practices. MIS Quarterly. 1996;20(2):167-96.

2. Stewart KA, Segars AH. An Empirical Examination of the Concern for Information Privacy Instrument. Information Systems Research. 2002;13(1):36-49. doi: 10.1287/isre.13.1.36.97.

3. Malhotra NK, Kim SS, Agarwal J. Internet users' information privacy concerns (IUIPC): The construct, the scale, and a causal model. Information systems research. 2004;15(4):336-55.

4. Xu H, Gupta S, Rosson MB, Carroll JM, editors. Measuring mobile users' concerns for information privacy. 33rd International Conference on Information Systems (ICIS 2012); 2012; Orlando, FL.

5. Choi Y, Zhunis A, Dong W, Seering J, Park S, Cha M, et al. Privacy disclosure to large language models: A large-scale study on awareness, benefits, and concerns in health contexts across three countries. Computers in Human Behavior Reports. 2025;20:100841. doi: 10.1016/j.chbr.2025.100841.

6. Liu Z, Zou W, Lin C. Exploring the influence of privacy concerns, AI literacy, and perceived health stigma on AI chatbot use in healthcare: An uncertainty reduction approach. Patient Education and Counseling. 2025;140:109271. doi: 10.1016/j.pec.2025.109271.

7. Liu Z, Hu L, Zhou T, Tang Y, Cai Z. Prevalence overshadows concerns? Understanding Chinese users' privacy awareness and expectations towards LLM-based healthcare consultation. 2025 IEEE Symposium on Security and Privacy (SP); San Francisco, CA. 2025. p. 2716-34.

8. Tran S, Lu H, Slaughter I, Herman B, Dangol A, Fu Y, et al., editors. Understanding Privacy Norms Around LLM-Based Chatbots: A Contextual Integrity Perspective. Proceedings of the AAAI/ACM Conference on AI, Ethics, and Society; 2025.
